# Using Gene Expression to Study Specialized Metabolism—A Practical Guide

**DOI:** 10.3389/fpls.2020.625035

**Published:** 2021-01-12

**Authors:** Riccardo Delli-Ponti, Devendra Shivhare, Marek Mutwil

**Affiliations:** School of Biological Sciences, Nanyang Technological University, Singapore, Singapore

**Keywords:** transcriptomics, co-expression, clustering, enrichment, online, metabolism

## Abstract

Plants produce a vast array of chemical compounds that we use as medicines and flavors, but these compounds’ biosynthetic pathways are still poorly understood. This paucity precludes us from modifying, improving, and mass-producing these specialized metabolites in suitable bioreactors. Many of the specialized metabolites are expressed in a narrow range of organs, tissues, and cell types, suggesting a tight regulation of the responsible biosynthetic pathways. Fortunately, with unprecedented ease of generating gene expression data and with >200,000 publicly available RNA sequencing samples, we are now able to study the expression of genes from hundreds of plant species. This review demonstrates how gene expression can elucidate the biosynthetic pathways by mining organ-specific genes, gene expression clusters, and applying various types of co-expression analyses. To empower biologists to perform these analyses, we showcase these analyses using recently published, user-friendly tools. Finally, we analyze the performance of co-expression networks and show that they are a valuable addition to elucidating multiple the biosynthetic pathways of specialized metabolism.

## Introduction

Despite the therapeutic and industrial potential of specialized plant metabolites (SM, also called secondary metabolites), their total chemical synthesis is often prohibitively expensive or even impossible due to their structural complexity (Chemler and Koffas, 2008). As a consequence, most of the SM are still extracted from their plant sources. The plant sources are often difficult to cultivate, resulting in the overharvesting of these species from the wild, as exemplified by firmoss (*Huperzia serra*), the pacific yew (*Taxus brevifolia*), and golden root (*Rhodiola rosea*; Busing et al., 1995; Lan et al., 2013). Furthermore, many valuable SM can be present at low concentrations in plants, precluding the production of these beneficial molecules in a cost-efficient manner. Consequently, large efforts are underway to understand the SM biosynthetic pathways, as these pathways can be engineered into more suitable microbial or plant hosts and further modified to produce novel, more potent compounds.

Despite the efforts to elucidate the plant SM biosynthetic pathways, very few pathways have been studied to completion, and even fewer have been transferred to heterologous hosts. A few examples include artemisinic acid (Paddon et al., 2013), the monoterpenoid indole alkaloids (Brown et al., 2015), and the benzylisoquinoline alkaloids (Thodey et al., 2014). This is a stark contrast to the >700 bacterial and fungal SM biosynthetic pathways that have been characterized and engineered (Cimermancic et al., 2014). There are two main reasons for this discrepancy between plants and microbes. Firstly, the enzymes biosynthesizing a SM in microbes are typically organized as biosynthetic gene clusters (BGCs), i.e., in a contiguous manner on chromosomes (Keller, 2019), which greatly simplifies the identification of the biosynthetic pathways. Conversely, in plants, the majority of SM pathways are not found in BGCs (Kliebenstein et al., 2012; Shi and Xie, 2014). However, nearly two dozen BGCs making defensive compounds have been functionally characterized, indicating that BGCs can be used to predict plant SM pathways in some cases (Nützmann et al., 2016; Kautsar et al., 2017; Tohge and Fernie, 2020). Secondly, in contrast to microbes, biosynthetic enzymes in plants comprise multiple, large gene families (e.g., cytochrome p450 family can comprise up to 1% of all plant genes; Mizutani and Ohta, 2010), complicating the assignment of an enzyme to a correct pathway based on genomic approaches alone. Consequently, many plant SM pathways, such as artemisinin, salicin, and taxol, have been elucidated by time-consuming and complex experimental approaches such as activity-guided fractionation, where the relevant enzyme is purified by multiple rounds of activity-guided fractionation, and identified by a proteomic approach, such as mass spectrometry.

Fortunately, the last decade has seen the emergence of novel methods in the area of genomics, transcriptomics, proteomics, metabolomics, synthetic biology, and gene function prediction, which has fueled the identification of SM biosynthetic pathways (Jacobowitz and Weng, 2020; Mutwil, 2020). These additional approaches provide multipronged sources of information to predict the identity of the enzymes making a given SM, allowing rapid *de novo* biosynthetic pathway prediction in nonmodel plants (Torrens-Spence et al., 2016). These predictions can then be rapidly tested by synthesizing codon-optimized cDNA of the putative enzyme and expressed in a laboratory microbe or a more suitable plant, such as *Nicotiana benthamiana* [please see the excellent review on these approaches in Jacobowitz and Weng, 2020)]. The various computational approaches comprising sequence similarity, Quantitative Trait Loci/Genome-Wide Association Studies (QTL, GWAS), phylogenetic profiling, and machine learning have been extensively reviewed elsewhere (Jacobowitz and Weng, 2020; Mutwil, 2020).

This review focuses on gene expression and co-expression networks as tools to uncover SM biosynthetic pathways. To showcase some of the analyses, we dissect biosynthetic pathways of sporopollenin, lignin, cutin, and suberin. We also discuss another important but overlooked property of gene expression and co-expression analyses: the ability to identify transcription factors and transporters as additional genes involved in the metabolites’ regulation and biosynthesis. Finally, we discuss some of the caveats typical for these analyses.

## Correlating Metabolite Presence and Gene Expression

Specialized metabolites often show a restricted presence in only a few organs, tissues, and cell types (Li et al., 2016), and can be extensively regulated by environmental factors (e.g., pathogen attack, UV-B light; Li et al., 2015; Tohge et al., 2016). For example, plant defense metabolites are frequently present in specialized tissues/cell types to minimize autotoxicity in the surrounding tissues and/or to maximize the effectiveness of these metabolites toward the spatially specific attacks of the aggressors (Schilmiller et al., 2010; Tissier, 2012). Of the 895 non-redundant metabolite spectra from different tissues of *Nicotiana attenuata*, 595 (63%) displayed tissue-specific expression, showing that SM often have organ- and tissue-specific gene expression (Li et al., 2016). Intuitively, the biosynthetic enzymes and their mRNAs should only be present in the cells where the metabolite is made. This assumption can be exploited to identify the biosynthetic genes by correlating gene expression and metabolite levels. This assumption fails for cases where the site of metabolite biosynthesis and accumulation differs, as exemplified by nicotine, which is biosynthesized in roots by root-specific enzymes and exported to leaves (Katoh et al., 2005; Tan et al., 2020). However, this simple yet powerful analysis has been successfully applied to unravel biosynthetic pathways of modified fatty acids in tomato (Jeon et al., 2020) and colchicine in *Gloriosa superba* (Nett et al., 2020).

**FIGURE 1:**
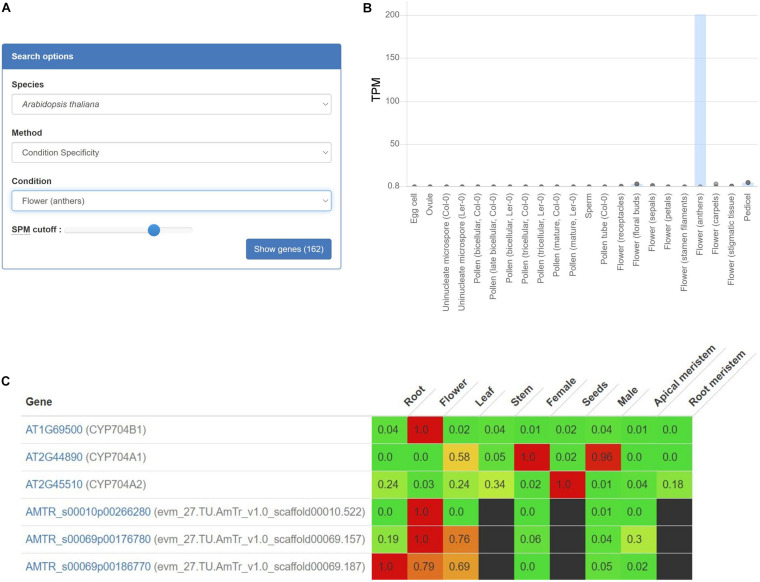
Expression profiles of *AT1G69500* (*CYP704B1*), a cytochrome P450 involved in pollen exine formation. **(A)** User interface of the CoNekT tool used to identify organ-specific genes. **(B)** The plot shows the expression of *CYP704B1* in *Arabidopsis*. The various organs and tissues are shown on the *x*-axis, while the *y*-axis indicates expression levels as Transcripts Per Million (TPM). The gray points indicate the minimum and maximum expression. **(C)** Expression profiles of *CYP704B1* and its orthologs in seed plants. Green and red color indicate low and high expression, respectively, while black cells indicate missing gene expression data. The figure contains expression profiles of genes from *Arabidopsis* (AT) and *Amborella* (AMTR). The CoNekT platform groups various tissues (e.g., petals, anthers, and pistils) from an organ (e.g., flower) into one category. Each cell contains the average expression values of samples from the organ. For brevity, only genes from *Amborella* and *Arabidopsis* are shown.

To exemplify how gene expression specificity can uncover a biosynthetic pathway, we use the CoNekT online tool^1^ (Proost and Mutwil, 2018) to analyze pollen exine biosynthesis. Pollen exine is an outermost protective layer of pollen grains, and consists of the insoluble sporopollenin biosynthesized in anthers (Hsieh and Huang, 2007). Thus, by identifying other genes with anther-specific gene expression, we should find the exine biosynthetic genes. To perform this analysis, we navigated to the “Tools/Find Specific Profiles,” selected *Arabidopsis* and “Flowers (anthers)” as the target species and tissue, which revealed 162 genes with another-specific expression (Figure 1A and Supplementary Table 1). As expected, these genes show exclusively anther-specific expression profiles (Figure 1B). Among these genes, we found numerous genes with unknown function, transcription factors, lipid transfer proteins, and several genes implicated in sporopollenin biosynthesis (Table 1). Notably, the analysis can reveal non-enzymatic genes essential for the functioning of the pathways, such as transporters needed for shuttling of the metabolite precursors (ABCG26) and transcription factors controlling the expression of the pathway (MYB103).

**TABLE 1:** Annotation of anther-specific genes involved in sporopollenin biosynthesis.

Gene ID	Symbol	Annotation	Function
*AT3G13220*	*ABCG26, WBC27*	ABC-2 type transporter family protein	Polyketide export Quilichini et al., 2014
*AT1G62940*	*ACOS5*	Acyl-CoA synthetase 5	Sporopollenin monomer biosynthesis de Azevedo Souza et al., 2009
*AT4G34850*	*LAP5*	Chalcone and stilbene synthase family protein	Biosynthesis of pollen fatty acids and phenolics found in exine Dobritsa et al., 2010
*AT1G02050*	*LAP6*	Chalcone and stilbene synthase family protein	Biosynthesis of pollen fatty acids and phenolics found in exine Dobritsa et al., 2010
*AT1G01280*	*CYP703A2, CYP703*	Cytochrome P450, family 703, subfamily A, polypeptide 2	Biosynthesis of medium-chain hydroxy fatty acids Morant et al., 2007
*AT1G69500*	*CYP704B1*	Cytochrome P450, family 704, subfamily B, polypeptide 1	Biosynthesis of long-chain fatty acids Dobritsa et al., 2009
*AT5G56110*	*MYB103, AtMYB103, ATMYB80, MS188*	Myb domain protein 103	Tapetum and exine development Zhang et al., 2007

Expression profiles can also identify functionally equivalent genes across species. For example, gene *AT1G69500* (*CYP704B1*) is a cytochrome P450 long-chain fatty acid {omega}-hydroxylase essential for pollen exine formation (Dobritsa et al., 2009). Cytochrome P450 genes comprise one of the largest gene families that catalyze various metabolic reactions (Xu et al., 2015). Due to numerous duplications, it can be challenging to identify P450 genes involved in sporopollenin biosynthesis in other plants. However, since all sporopollenin-specific P450s are also likely expressed in anthers in other species, we can use gene expression to identify the relevant genes. We used CoNekT to compare expression profiles of the orthogroup containing *AT1G69500* and 78 other land plant-specific genes (https://evorepro.sbs.ntu.edu.sg/family/view/131885, click on “row-normalized” to view expression). As expected, *AT1G69500* is expressed specifically in flowers (CoNekT groups components of an organ into one category), while for *Amborella trichopoda*, only *AMTR_s00010p00266280* is showing a similar expression pattern, suggesting that *AT1G69500* and *AMTR_s00010p00266280* are functionally equivalent (Figure 1C).

## Using Guide Genes to Identify Biosynthetic Pathways

To uncover the other biosynthetic pathway components, it is possible to identify other genes with a similar expression profile if at least one of the biosynthetic enzymes is known (Usadel et al., 2009; Serin et al., 2016). This assumption is based on the observation that genes with similar expression patterns across organs, developmental stages, and biotic and abiotic perturbations tend to be involved in related biological processes. Identification of genes with similar profiles can be made by calculating all possible pairwise comparisons of gene expression profiles using different similarity metrics (e.g., Pearson Correlation Coefficient, Mutual Rank, and Highest Reciprocal Rank), across tens to thousands of gene expression measurements captured by microarrays or RNA sequencing (RNA-seq; Usadel et al., 2009; Mutwil et al., 2010; Aoki et al., 2016).

**FIGURE 2:**
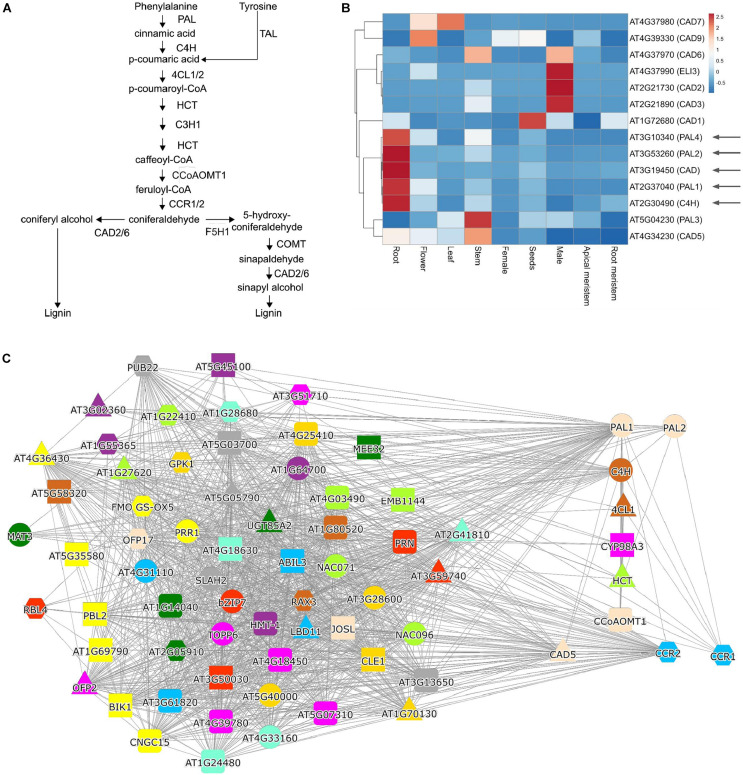
Analysis of lignin biosynthesis with expression clustering and co-expression network approaches. **(A)** Biosynthetic pathway of lignin. **(B)** Hierarchical clustering of *PALs*, *C4H*, and *CAD* genes. The red and blue colors indicate high and low expression in a given organ, respectively. **(C)** Co-expression network of *PAL1*. Nodes represent genes, gray edges connect co-expressed genes, while node colors indicate orthogroups of the gene families. The red square in **(B,C)** indicate genes known to be involved in lignin biosynthesis.

The identification of these transcriptionally co-regulated (co-expressed) genes has been successfully used to further complete various metabolic pathways, such as protolimonoids from *Azadirachta indica* (Hodgson et al., 2019), vinblastine from Madagascar periwinkle (Caputi et al., 2018), etoposide glycone from *Podophyllum hexandrum* (Lau and Sattely, 2015), and the seco-iridoid pathway from *Catharanthus roseus* (Miettinen et al., 2014), to name a few recent examples. The identification of the co-expressed genes can be performed in three ways, by a: (i) co-expression list analysis, (ii) hierarchical clustering of expression profiles, or (iii) co-expression networks. To exemplify how these analyses can be performed and interpreted, we use the classical example of lignin biosynthesis, which requires multiple steps to convert phenylalanine to various lignin precursors (Figure 2A; Sibout et al., 2017).

## Uncovering Functionally Related Genes by the Co-Expression List Analysis

The co-expression list analysis is typically a “one versus all” analysis, where the expression profile similarity of one gene is compared to expression profiles of all genes, and the resulting list is sorted according to a similarity metric, such as the Pearson Correlation Coefficient (PCC; Usadel et al., 2009). Typically, this analysis is used to uncover unknown components of a biological process (Brown et al., 2005; Persson et al., 2005). Since the list is sorted according to expression profile similarity, the most relevant genes are found on top of the list, and typically top 50 genes are investigated (Aoki et al., 2016; Proost and Mutwil, 2018). The analysis of phenylalanine ammonia-lyase 1 (*PAL1*), which is the first enzyme in the phenylpropanoid pathway needed for lignin biosynthesis (Figure 2A), revealed several known players, such as *C4H*, *PAL2*, *CYP98A3*, *CCR1*, *CCR2*, *4CL*, and *HCT* (Table 2 and Supplementary Table 2). It is important to note that the list does not contain all of the lignin biosynthetic enzymes, showing that co-expression is not always guaranteed to retrieve all relevant genes. To uncover the pathway’s missing members, we recommend using other known members of the pathway as a query and collate the results.

**TABLE 2:** Co-expression list of PAL1.

Sequence	Annotation	PCC	Function
AT2G37040	Phenylalanine ammonia-lyase 1 ATPAL1, PAL1	1.0	Phenylpropanoid pathway entry Cochrane et al., 2004
AT2G30490	Cinnamate-4-hydroxylase ATC4H, CYP73A5, REF3, C4H	0.836494	*Trans*-4-coumarate biosynthesis Schilmiller et al., 2009
AT3G53260	Phenylalanine ammonia-lyase 2 ATPAL2, PAL2	0.806119	Phenylpropanoid pathway entry Cochrane et al., 2004
AT2G40890	Cytochrome P450, family 98, subfamily A, polypeptide 3 CYP98A3	0.647512	3’-hydroxylation of *p*-coumaric esters Schoch et al., 2001
AT1G80820	Cinnamoyl-Coa reductase CCR2, ATCCR2	0.624933	Cinnamaldehyde biosynthesis Lacombe et al., 1997
AT1G51680	4-coumarate:CoA ligase 1 4CL1, AT4CL1, 4CL.1	0.609514	CoA thiol ester biosynthesis Ehlting et al., 1999
AT5G48930	Hydroxycinnamoyl-CoA shikimate/quinate hydroxycinnamoyl transferase HCT	0.589046	Hoffmann et al., 2004
AT1G15950	Cinnamoyl-Coa reductase 1 CCR1, IRX4, ATCCR1	0.525528	Cinnamaldehyde biosynthesis Lacombe et al., 1997

*For brevity, only the known participants of the lignin biosynthesis pathway are shown.*

## Hierarchical Clustering Analysis

Hierarchical clustering of expression profiles is a “many versus many” analysis, where the selected genes are grouped into clusters defined by expression profile similarity. These clusters are then visually analyzed to identify genes containing the known components of a pathway and exclude genes that are not part of these clusters. Typically, this analysis is used when the list of candidate genes is extensive and needs to be reduced. This approach has been used in identifying P450 enzymes important for protolimonoid synthesis (Hodgson et al., 2019) and components of etoposide aglycone biosynthesis (Lau and Sattely, 2015). To exemplify a clustering analysis, we selected four PAL gene family members, *ATC4H*, and nine members of the CAD family. We entered the 14 (*AT2G37040, AT3G10340, AT3G53260, AT5G04230, AT2G30490, AT1G72680, AT2G21730, AT2G21890, AT3G19450, AT4G34230, AT4G37970, AT4G37980, AT4G37990*, and *AT4G39330*) genes into the ‘‘Tools/Heatmap/Comparative’’^2^, which revealed the expression profiles of these genes in organs of *Arabidopsis*. The resulting heatmap was pasted into the ClustVis web-tool^3^ (Metsalu and Vilo, 2015) and used to perform hierarchical clustering. The heatmap revealed that *PAL1,2* and *4* are clustering with *C4H* and *CAD*, but, e.g., not with *PAL3*, which has not been implicated in lignin biosynthesis (Figure 2B). The heatmap can also indicate where a given cluster is expressed, showing that the lignin cluster has the highest expression in roots. In contrast, the other major cluster containing *CAD2, 3, 6*, and *ELI3* are expressed in male organs (comprising pollen and sperm, Figure 2B). Thus, the clustering analysis can reveal functionally related genes and indicate the organs and tissues where these genes are likely active.

## Co-Expression Network Analysis—Searching With a Query Gene

Co-expression networks can be used in “many versus many” (when used with one query gene) or “all versus all” (when used with co-expression clusters) type of analyses. In co-expression networks, nodes (or vertices) represent genes, and edges (or links) connect genes that display similar expression profiles (Lee et al., 2004; Usadel et al., 2009; Serin et al., 2016). While the networks are different from co-expression lists (lists are ordered while networks are not) and hierarchical clustering (networks are unordered and typically do not indicate the expression patterns of genes), when used with one query gene, the networks provide the same information: the identity of functionally-related genes. To exemplify a typical network analysis, we used *PAL1^4^*, which similarly to the co-expression list (Table 2), retrieved several, but not all, known participants of lignin biosynthesis (Figure 2C).

In contrast to lists and hierarchical clustering approaches, networks can convey additional information with node and edge colors. For example, CoNekT uses different node colors and shapes to indicate gene families (see text footnote 4; Proost and Mutwil, 2018), while ATTED-II^5^ (Aoki et al., 2016), and GeneMANIA^6^ (Warde-Farley et al., 2010) use edge styles to indicate different types of functional relationships between genes (e.g., co-expression, protein-protein interactions). Modern tools provide interactive networks, where the nodes can be moved, colored by different criteria (e.g., by organ-specific expression or gene family membership), allowing adjusting the networks to convey the desired information better.

## Identifying Functionally Related Genes by Custom Network Analysis

While a typical genome-wide co-expression network typically contains tens of thousands of nodes (genes) and millions of edges (connections), a typical user is only interested in a particular part of the network representing a biological process of interest. Since functionally related genes tend to be connected, the network can be used to uncover functional clusters of genes. Conceptually, the analysis is similar to hierarchical clustering (Figure 2B), but instead of clades, the functionally related genes are connected by edges.

While most current studies focus on uncovering the enzymes constituting a biosynthetic pathway, non-enzymatic genes are also crucial for SM’s efficient biosynthesis. For example, gliotoxin biosynthesis in fungi Aspergillus requires a gliotoxin efflux pump that removes the harmful metabolite from the cellular environment. At the same time, another enzyme modifies it to a less toxic form (Dolan et al., 2015). Furthermore, up to 50% of BGCs in fungi also contain transcription factors that positively regulate the corresponding pathway (Brown et al., 2015). In plants, we observed that relevant transcription factors and transporters can be co-expressed with the pathways they regulate and participate in, respectively. For example, we observed ABCG26, a polyketide transporter needed for exine biosynthesis in *Arabidopsis* (Table 1), and in *Brachypodium distachyon* various other transporters and transcription factors important for cellulose biosynthesis (Sibout et al., 2017), artemisinin biosynthesis in *Artemisia annua* (Tan and Mutwil, 2019) and nicotine biosynthesis in *Nicotiana tabacum* (Tan et al., 2020). Thus, co-expression analysis is uniquely positioned to reveal non-enzymatic components essential for the efficient functioning of metabolic pathways.

**FIGURE 3:**
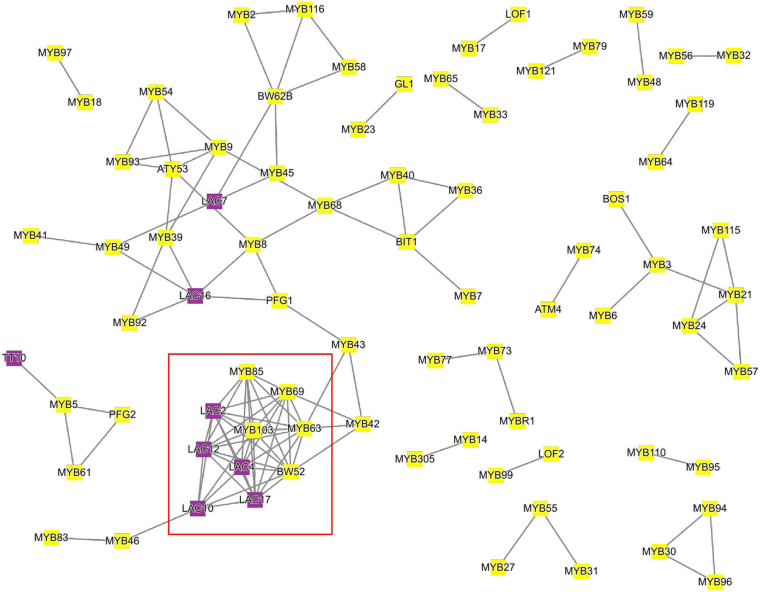
Co-expression network of *Arabidopsis* laccases and MYB transcription factors. Nodes represent genes, gray edges connect co-expressed genes, while node colors indicate MYBs (yellow) or laccases (purple). The red square indicates MYBs and laccases implicated in lignin biosynthesis. For brevity, only genes that are connected to at least one other gene are shown.

To demonstrate how this analysis can be performed, we tested which MYB transcription factors are co-expressed with lignin biosynthesis-related laccases (LAC) in *Arabidopsis* (Figure 3). To this end, we used as input the 11 LAC genes^7^, together with 122 MYB transcription factors^8^ into the ‘‘Tools\Create custom network’’ tool^9^. We observed the association of laccases necessary for lignin biosynthesis in the secondary cell wall (*LAC2*, *LAC4*, and *LAC17*; Berthet et al., 2011; Khandal et al., 2020) with MYBs controlling lignin biosynthesis (MYB103, MYB85, MYB63, and MYB52; Zhou et al., 2009; Cassan-Wang et al., 2013; Öhman et al., 2013; Geng et al., 2020). Interestingly, we also observed the association of MYB5, which controls seed coat development (Li et al., 2009) to TT10, which is essential for flavonoid biosynthesis in the seed coat (Pourcel et al., 2005). Since CoNekT allows quick retrieval of gene families representing different gene functions, we envision that this functionality can be used to rapidly highlight transcription factors, transporters, and other genes necessary for the biosynthetic pathways.

## Searching Co-Expression Clusters for Enriched Biosynthetic Pathways

One of the significant advantages of co-expression networks is the availability of graph-theoretical methods to define co-expression clusters, i.e., groups of genes with similar expression profiles (Ronan et al., 2016). This simplifies gene expression data analysis, as clustering typically assigns tens of thousands of genes into hundreds of co-expression clusters. The clusters can then be compared to identify groups with similar functions across species (Heyndrickx and Vandepoele, 2012) or duplicated modules within species (Ruprecht et al., 2016). Furthermore, the clusters’ biological function can be elucidated by identifying enriched Gene Ontology or MapMan terms (Sibout et al., 2017; Ferrari et al., 2020).

To demonstrate how searching for functionally enriched clusters can be used to generate novel insights, we selected cutin and suberin as an example. Cutin and suberin are lipid biopolyester components of the cell walls important for desiccation tolerance (Philippe et al., 2020). To identify a module biosynthesizing cutin in *Arabidopsis*, we navigated to the “Tools/Find enriched clusters,” entered “cutin biosynthesis” under GO search box, and clicked “Show clusters.” This revealed three clusters significantly (*p*-value < 0.05) enriched for genes known to be involved in cutin biosynthesis in *Arabidopsis*, and we clicked on cluster 26. The page dedicated to the cluster provides information about the average expression profile of the genes in the cluster, the identity of the genes, and functional enrichment analysis^10^. The “Similar clusters” table found on the cluster page also contains the identity of similar clusters across and within species (similarity is defined by Jaccard index between cluster gene families; Proost and Mutwil, 2018), allowing an easy way to identify conservation and duplication of biosynthetic pathways (Ruprecht et al., 2016). Interestingly, we observed that cluster 206 from *Arabidopsis* is most similar to cutin cluster 26, indicating that the cutin cluster has been duplicated to biosynthesize a cutin-like polymer in another organ or tissue.

**FIGURE 4:**
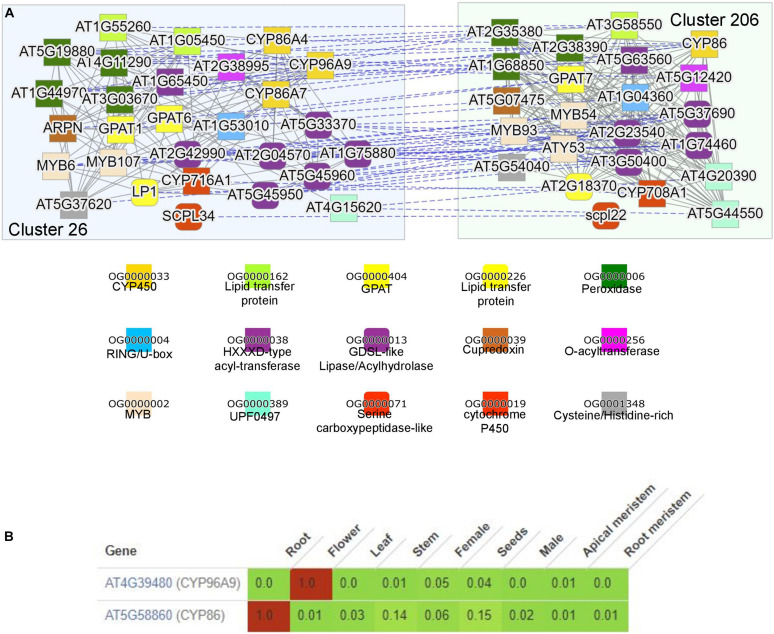
Comparison of the duplicated clusters involved in suberin and cutin biosynthesis. **(A)** Co-expression networks of module 26 (blue, left) and 206 (green, right) from *Arabidopsis thaliana*. The annotation of the colored shapes of the gene families (given as orthogroups OG) is shown below. **(B)** Expression profiles of *At4g39480* from cluster 29 and *At5g58860* from cluster 206. Green and red colors indicate low and high expression, respectively. The expression values are scaled by dividing each row by the maximum expression found in the row. The tool used is available at the “Tools/Generate heatmap/Comparative” on CoNekT’s homepage.

By clicking on the “Compare” button next to the duplicated cluster 206, the two clusters are visualized (Figure 4A). The two clusters contain numerous gene families that have been implicated in the biosynthesis of cutin and suberin, comprising CYP450s, lipid transfer proteins, acyl-transferases, and *glycerol-3-phosphate acyltransferase* (*GPAT*; Philippe et al., 2020). Cutin is predominantly present in aerial organs, while suberin is mostly present in roots and seed coats (Philippe et al., 2020). In line with this, comparative expression profile analysis of two representative CYP450s revealed the expected expression of cluster 26 in flowers and cluster 206 in roots (Figure 4B). Interestingly, *MYB107* has been shown to regulate suberin biosynthesis (Gou et al., 2017), but is also found in the cutin cluster, suggesting that it might also have a role in cutin biosynthesis. We also observed numerous other gene families (e.g., cupredoxin, cysteine/histidine-rich, carboxypeptidases, and RING/U-box), which are not implicated in the biosynthesis of the polymers. However, since these gene families are present in both clusters, they are likely involved in some aspect of their biosynthesis.

To conclude, enriched cluster analysis can reveal the clusters comprising various biosynthetic pathways. The conserved or duplicated modules can identify the conserved (i.e., likely relevant) genes found in the pathways.

## Performing Your Own Analysis With Existing Tools or Your Own Data

While the above analyses exemplified how CoNekT can be used to study SM, multiple online tools are available, such as ATTED-II (Aoki et al., 2016), CoNekT (Proost and Mutwil, 2018), PlaNet (Mutwil et al., 2011), ePlant (Waese et al., 2017), and PlantGenIE (Sundell et al., 2015) reviewed in Rao and Dixon (2019). These tools are preloaded with expression data from tens of plants of agricultural and evolutionary interest (Table 3). Still, there are >200,000 RNA-seq experiments publicly available for >100 species from the plant kingdom^11^, providing an excellent opportunity to study the biosynthetic pathways of SM. Furthermore, as RNA-seq analysis is becoming more affordable and accessible, numerous studies nowadays generate and analyze their own RNA-seq data to prioritize genes for functional analysis. To perform such an analysis, we need (i) coding sequence (CDS) file, (ii) gene expression data, and (iii) gene expression similarity analysis.

**TABLE 3:** Online tools allowing expression profiles and co-expression network analysis.

	ATTED (https://atted.jp/)	ePlant (http://bar.utoronto.ca/)	PlantGenIE (https://plantgenie.org/)	PlaNet (www.gene2function.de)	CoNekT (www.evorepro.plant.tools)
*Amborella trichopoda*	N	N	N	N	Y
*Arabidopsis thaliana*	Y	Y	Y	Y	Y
*Brassica rapa*	Y	N	N	N	N
*Chlamydomonas reinhardtii*	N	N	N	Y	Y
*Cyanophora paradoxa*	N	N	N	N	Y
*Eucalyptus grandis*	N	N	Y	N	N
*Ginkgo biloba*	N	N	N	N	Y
*Glycine max*	Y	N	N	Y	N
*Marchantia polymorpha*	N	N	N	N	Y
*Medicago truncatula*	Y	N	N	Y	N
*Oryza sativa*	Y	Y	N	Y	Y
*Physcomitrella patens*	N	N	N	Y	Y
*Picea abies*	N	N	Y	N	Y
*Populus trichocarpa*	Y	N	Y	Y	N
*Selaginella moellendorffii*	N	N	N	N	Y
*Solanum lycopersicum*	Y	N	N	N	Y
*Vitis vinifera*	Y	N	N	N	Y
*Zea mays*	Y	Y	N	N	Y

*Only tools that are preloaded with co-expression networks for more than two plants are shown.*

The CDS file contains the transcript sequences the RNA-seq data should be mapped too. A CDS file can be typically retrieved from a public database, such as the EnsemblGenone^12^ or Phytozome^13^, or the genome release paper, if available. If no genome is available, RNA sequencing data can be used for *de novo* assembly. Best-performing transcriptome assemblers are typically able to retrieve >70% of the expected gene space (Hölzer and Marz, 2019). Indeed, elucidation of biosynthetic pathways without a reference genome successfully revealed steps in colchicine alkaloid (Nett et al., 2020) and protolimonoid biosynthesis (Hodgson et al., 2019), showing that the RNA-seq data can be used as an acceptable source for CDS. Comparison of 10 transcriptome *de novo* assembly tools across nine RNA-seq datasets spanning different kingdoms of life showed that Trinity, SPAdes, and *Trans*-ABySS consistently show the highest performance in reconstructing the coding sequences (Hölzer and Marz, 2019), where Spades has the easiest setup, user-friendliness, and lowest memory usage and runtime.

The gene expression data is used to reveal the functional associations between the genes. While as few as eight samples can be sufficient to identify relevant members of a metabolic pathway (Nett et al., 2020), the expression data should ideally capture organs/tissues which show contrasting levels of the metabolite of interest. For example, among the four organs of *G. superba* (leaf, stem, rhizome, and root), colchicine alkaloids showed the highest accumulation in the rhizome, which allowed the authors to elucidate most of the pathway by identifying rhizome-specific genes by clustering analysis. In another study, the authors took advantage of highly specific induction of falcarindiol biosynthesis by pathogen elicitors and identified six acetyltransferases that were upregulated upon treatment (Jeon et al., 2020). Conversely, the lignin (Figures 2, 3), suberin, and cutin (Figure 4) examples from *Arabidopsis* use one dataset containing hundreds of publicly available RNA-seq experiments that captures different organs, developmental stages, and growth conditions. This comprehensive dataset can thus be potentially used to identify all *Arabidopsis* biosynthetic pathways, as long as the dataset captures the organs where a given pathway is expressed. We have developed a user-friendly, cloud computing pipeline, LSTRaP-Cloud^14^, that provides tools to download and quality-control publicly available gene expression data and to perform co-expression list and co-expression network guide gene analyses (Tan et al., 2020). Alternatively, Curse can perform these analyses on the user’s computer and allow the semi-automated annotation of the RNA-seq experiments^15^ (Vaneechoutte and Vandepoele, 2019).

The gene expression similarity analysis is used to identify genes with similar expression patterns, which is the basis for identifying functionally-related genes. If one or multiple guide genes are known, we recommend the co-expression list approach (Table 2), which can be performed by the LSTRaP-Cloud or Curse. To identify gene clusters containing known participants of the pathway of interest, clustering-based analyses of the expression matrix (Table 1 and Figure 2B) can be done with the ClustVis web-tool^16^ (Metsalu and Vilo, 2015). Alternatively, CoExpNetViz allows the upload and co-expression analysis of the user’s gene expression data^17^ (Tzfadia et al., 2016), and CoNekT provides source code and instructions to set up a stand-alone database^18^ (Proost and Mutwil, 2018).

## Is Co-Expression a Silver Bullet in Biosynthetic Pathway Discovery? Not Quite

The above examples demonstrate that gene expression and co-expression analyses are valuable additions to the SM pathway discovery toolbox. However, as with many guilt-by-association methods, we often observe many missing enzymes (false negatives) and irrelevant genes (false positives). This is exemplified by Figure 2C, where, e.g., COMT enzyme is not detected (false negative) and where a large number of seemingly irrelevant genes are found in the lignin biosynthesis network (false positive).

**FIGURE 5:**
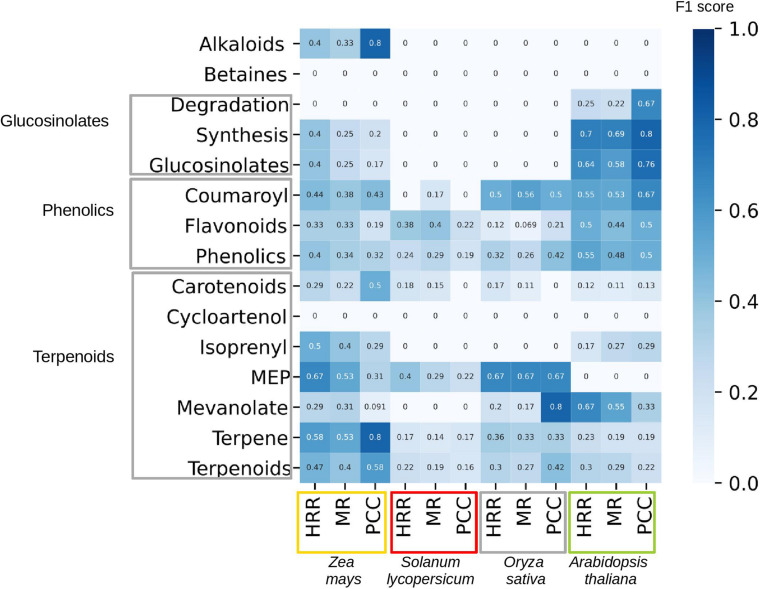
Performance of co-expression networks in predicting correct enzymes in specialized metabolism. The rows contain different SM classes, as defined by MapMan, while the columns contain four plants: maize (*Zea mays*, orange box), tomato (*Solanum lycopersicum*, red box), rice (*Oryza sativa*, gray box), and *Arabidopsis thaliana* (green box). For each species, we calculate the performance for three networks, based on: Higest Reciprocal Rank (HRR), Mutual Rank (MR), and Pearsson Correlation Coefficient (PCC). The shade of the cells and the cell numbers correspond to F1 score (harmonic mean of precision and recall), which ranges from 0 (poor performance of prediction or too few genes associated to a specific pathway to perform a prediction) to 1 (perfect performance).

To gage the co-expression networks’ performance in identifying SM genes, we tested three network construction methods (PCC, HRR, and MR) from four different species (*Zea mays, Solanum lycopersicum, Oryza sativa, and Arabidopsis thaliana*). The used networks are based on gene expression data representing all major plant organs at different developmental stages (Julca et al., 2020). We analyzed 15 different secondary metabolic pathways associated with alkaloids, betaines, glucosinolates, phenolics, and terpenoids (Figure 5). We then predicted genes that are involved in each of the 15 pathways, by using a network neighborhood approach (Hew et al., 2020), and the F1 score to see how known members of each pathway could be correctly classified by each of the networks. We observed a complex interplay between the different metabolic pathways and species. For example, the performance of the networks was higher in *Arabidopsis* than tomato for nearly all pathways, while, e.g., terpene pathway could be more readily predicted in maize than *Arabidopsis* (higher scores in the latter plant), for all three types of networks (HRR, MR, and PCC). Conversely, methylerythritol 4-phosphate (MEP) pathway could not be predicted at all in *Arabidopsis* (F1 score 0 for all networks). These results indicate that co-expression networks can show unpredictable performance when predicting SM pathways, and more research is needed to understand which conditions would result in best performance (quantity and quality of the expression data, the network construction methods).

## Conclusion and Future Perspectives

Gene expression and co-expression network analyses are valuable, unique tools to unravel the biosynthetic pathways of specialized metabolism. The expression-based analyses’ versatility allows shortlisting of gene candidates with even a few RNA sequencing samples (Nett et al., 2020) or elucidation of multiple pathways with one large expression dataset (Figures 1–4). We find ourselves in the log phase of metabolic pathway discovery as open-source online tools are publicly available (e.g., https://github.com/tqiaowen/LSTrAP-Cloud) and repositories are brimming with gene expression data for hundreds of plant species.

In addition to uncovering the enzymes underpinning the various metabolic pathways, the co-expression networks present two exciting, novel opportunities. Firstly, these analyses can reveal non-enzymatic components of the pathways, such as transporters and transcription factors (Table 1 and Figure 3). The transcription factors are especially exciting, as changing their expression can alter the whole pathway’s activity and cause dramatic changes in metabolite levels (Zhao and Dixon, 2011). Secondly, the networks can serve as top-down tools to uncover new pathways by identifying novel clusters of connected genes. For example, the analysis investigating the functional association between MYB transcription factors and laccases (Figure 3) can be repurposed to study associations between all enzymes in an organism. The analyses discussed in this review can and should be supplemented with other omics-based inference methods to pave the way for more nutritious, resilient crops, and the development of novel medicines.

## Author Contributions

RD-P contributed to the co-expression performance analysis. DS helped with the literature summary. MM designed the review. All authors helped with the manuscript.

## Conflict of Interest

The authors declare that the research was conducted in the absence of any commercial or financial relationships that could be construed as a potential conflict of interest.

## Footnotes

1 https://evorepro.sbs.ntu.edu.sg/search/specific/profiles

2 https://evorepro.sbs.ntu.edu.sg/heatmap/

3 https://biit.cs.ut.ee/clustvis/

4 https://evorepro.sbs.ntu.edu.sg/network/graph/3767

5 https://atted.jp/locus/?gene_id=818280

6 https://genemania.org/search/arabidopsis-thaliana/pal1

7 https://evorepro.sbs.ntu.edu.sg/family/view/115

8 https://evorepro.sbs.ntu.edu.sg/family/view/3

9 https://evorepro.sbs.ntu.edu.sg/custom_network/

10 https://evorepro.sbs.ntu.edu.sg/cluster/view/212

11 https://www.ncbi.nlm.nih.gov/sra/

12 https://plants.ensembl.org/index.html

13 https://phytozome.jgi.doe.gov/pz/portal.html

14 https://github.com/tqiaowen/LSTrAP-Cloud

15 http://bioinformatics.psb.ugent.be/webtools/Curse/

16 https://biit.cs.ut.ee/clustvis/

17 http://bioinformatics.psb.ugent.be/webtools/coexpr/index.php

18 https://github.com/sepro/CoNekT

## Supplementary Material

Supplementary Table 1 Anther-specific genes retrieved from CoNekT. The genes shown in Table 1 are indicated by bold letters.

Supplementary Table 2 PAL1-coexpressed genes retrieved from CoNekT. The genes shown in Table 2 are indicated by bold letters.
